# Passive biaxial mechanical properties of sheep myocardium

**DOI:** 10.3389/fbioe.2025.1549829

**Published:** 2025-03-20

**Authors:** Thanyani Pandelani, Letlhogonolo Semakane, Makhosasana Msibi, Alex G. Kuchumov, Fulufhelo Nemavhola

**Affiliations:** ^1^ Department of Mechanical, Bioresources and Biomedical Engineering, Unisa, Pretoria, South Africa; ^2^ Department of Mechanical Engineering, Faculty of Engineering and the Built Environment, Durban University of Technology, Durban, South Africa; ^3^ Biofluids Laboratory, Perm National Research Polytechnic University, Perm, Russia; ^4^ Department of Computational Mathematics, Mechanics and Biomechanics, Perm National Research Polytechnic University, Perm, Russia

**Keywords:** myocardium, biaxial, anistropic, sheep, right ventricle (RV), left ventricle (LV)

## Abstract

**Introduction:** Myocardial infarction is a serious and potentially life-threatening condition that requires immediate medical intervention. The earlier help is provided, the less likely irreversible damage to the heart muscle will occur. Experimental investigation of myocardium behaviour is necessary for advanced numerical models to predict treatment outcomes.

**Methods:** The study investigates the mechanical characteristics of the sheep heart’s mid-wall, right and left ventricles using equi-biaxial mechanical testing. This method allows for studying the myocardium’s behaviour in multiple directions, specifically analyzing the mechanical stiffness and strain energy. Thirteen (13) sheep hearts were collected from a local abattoir, and ten (10) of them were prepared and subjected to equi-biaxial mechanical tests under physiological conditions. This was to ensure that hearts were healthy to minimise the variability in mechanical properties of the myocardium. The study measured stress-strain relationships in both the longitudinal and circumferential directions for the right ventricle (RV), left ventricle (LV), and mid-wall septum (MDW). To minimize viscoelastic effects, the preconditioning protocol involved cyclic loading of 10 cycles before testing.

**Results and discussion:** Results indicated distinct mechanical properties between the chambers, with the RV showing higher strain energy storage and compliance in the circumferential direction than the LV. To minimize viscoelastic effects, the preconditioning protocol involved cyclic loading of 10 cycles before testing. Stress-strain behaviour exhibited nonlinear characteristics, with variability between samples. Stored strain energy values of linear elastic region for left ventricle were 7.317 kJ and 6.67 kJ in longitudinal and circumferential directions, respectively. The elastic modulus was determined from the linear elastic region for each heart wall specifically, from 16% to 40% strain for LV, MDW, and RV. The toe region peak stresses were those corresponding to 16% strain for LV, MDW, and RV. The stresses at 40% strain were obtained from the closest strain level. Anisotropic effects of myocardium were exhibited. Thus, the study provides insights into the mechanical anisotropy of the myocardium and its relevance to ventricular function, offering important data for understanding heart tissue mechanics and modelling heart diseases.

## 1 Introduction

The heart has four chambers, namely two atria and two ventricles; the left ventricles (LV) is regarded as the main chamber (Genet et al., 2014; Laita et al., 2024). LV is vital; it pumps blood into the entire body (excluding the lungs) (Genet et al., 2014; Pandelani et al., 2023). The ventricular wall is made up of endocardium (innermost), myocardium (middle) and the epicardium being the outermost layer (Gültekin et al., 2016). The LV is thicker and has the largest volume compared to RV and MDW (Gültekin et al., 2016). The ability of ventricles to adjust during contraction in response to changes in afterload and preload, though not completely independently, is a key similarity between them (ventricles). Muscle fibers in the myocardium stretch during diastole (Bellofiore and Chesler, 2013).

There are challenges associated with the heart; the LV is more likely to fail and prone to diseases (Liu and Wang, 2019); this might be attributed to its functionality. The heart may become large and poorly contractile or thick and stiff depending on the type of failure associated with it (Genet et al., 2014). These changes may result in the malfunction of the constituent cells of the myocardium and the deterioration of the organ functionality (hemodynamic and mechanical) (Liu and Wang, 2019). Hence, the amount of blood pumped towards the body is reduced (Genet et al., 2014). Therefore, understanding the mechanical behaviour of these heart walls is vital in developing new therapies or enhancing the current therapies. Moreover, accurate constitutive models may be developed from the mechanical behaviour of these three walls (RV, MDW, and RV) (Nemavhola, 2021a). The shear properties of passive ventricular myocardium have been deducted utilising general constitutive models that incorporate elements of myocardial structure and data obtained from biaxial testing (Dokos et al., 2002).

The mechanical testing of ventricular samples from porcine, human, and canine under laboratory conditions has shown that when exposed to finite deformations, the myocardial tissue exhibits nonlinear hyperelastic and viscoelastic properties (Dokos et al., 2002; O’Shea and Attard, 2022; Sommer et al., 2015). Moreover, understanding the passive myocardium’s behaviour is vital to accurately model the heart’s mechanical function and gain a deeper understanding of cardiac mechanisms and diseases. This is also beneficial for advancing medical healthcare. For example, the diastolic mechanical properties of cardiac muscles are key in determining cardiac function, with specific passive myocardial stiffness contributing to diastolic heart failure conditions (Emig et al., 2021; Solovyova et al., 2016; Wang et al., 2016; Palit et al., 2018). Ventricular diastolic dysfunction in heart failure patients is linked to considerable mortality and morbidity. Therefore, the passive stiffness of the myocardium plays a crucial role in determining overall cardiac function (Krüger, 2024).

The myocardial tissue is not transversely isotropic, as evident from the simple shear tests conducted in various directions on the passive ventricular myocardium from porcine hearts by (Dokos et al., 2002). Due to its fluid content, the myocardium is considered incompressible (Liu et al., 2009). However, it undergoes ‘dynamic’ volumetric changes of up to 20% during the cardiac cycle. This behaviour is generally attributed to variations in coronary blood flow, which fluctuate by 20%–40% throughout the cardiac cycle (Laita et al., 2024). It is known that the LV and right ventricles (RV) have different morphology. However, less is known about RV failure, and whether these two chambers display similar mechanical behaviours in developing disease or ventricular dysfunction (Liu and Wang, 2019). LV has been the chamber of interest for many researchers due to its role. In the study by Gupta et al. of change in passive mechanical stiffness of myocardial tissue with aneurysm formation, they reported that for a 6-week infarcted heart, the ovine left ventricle sample was stiffer in the longitudinal direction. The modified Levenberg-Marquardt technique was utilised to fit the stress-extension data (Gupta et al., 1994).

Furthermore, in the study by Javani et al. of biomechanical properties and microstructure of heart chambers (a paired comparison study in an ovine), high strain energy storage was noticeable or attained in the left side of the heart for all the chambers than the corresponding chambers on the right side of the heart. In addition, the ventricles had the highest stored strain energy. This was attained through biaxial tests. The Fung-type strain energy function was utilised to fit the results obtained through biaxial mechanical tests (Javani et al., 2016).

Biaxial and uniaxial mechanical tests are the most general tests in investigating the mechanical properties of the ventricles. However, more comprehensive anisotropic mechanical behaviour measurements are attained through biaxial test (Liu and Wang, 2019). Moreover, due to the consideration of biological tissues as incompressible, the biaxial test is preferred to describe three-dimensional constitutive relations (Gupta et al., 1994).

Ghaemi et al. (2009) investigated and measured the passive mechanical properties of bovine myocardium through biaxial, uniaxial, and equiaxial mechanical testing. Hearts from eight (8) cattle ranging between 1 and 2 years old were considered. The samples were taken from the left ventricle free wall (LVFW), right ventricle free wall (RVFW), left ventricle mid-wall (LVMW), and the apex. A custom-designed biaxial tensile testing machine was employed, using T-shaped grips and a CCD camera to capture local strain at the centre of the specimens. Three different test protocols were performed; 1) tensile tests were conducted at stretch rates of 0.1, 0.2, 0.5, and 0.75 cm/s on LVFW, RVFW, LVMW, and apex samples (equibiaxial), 2) various stretch ratios were applied to study the cross-coupling of stresses between fiber and cross-fiber directions (Unequal biaxial), and 3) tensile tests were conducted with pulling in the fiber direction while the cross-fiber direction was fixed. For preconditioning, cycling loads were applied before the start of the actual tensile tests to reduce viscoelastic effects. The relationship between stress and stretch in the myocardium was non-linear, with the fiber direction being stiffer than the cross-fibre direction. For equi-biaxial testing, the maximum stress values observed in the fiber direction for LVFW, LVMW, RVFW, and apex at 0.1 cm/s and stretch rates are 10.93 and 11.32 kPa, 11–11.23 kPa, 10.75–10.89 kPa, and 10.69–10.79 kPa, respectively. For unequal biaxial testing, the cross-coupling effect between fiber and cross-fiber directions was observed. Cross-fiber stresses were observed to be increasing slightly when the fibres were stretched at varying ratios. For uniaxial testing, stress developed in the cross-fiber direction remained small even as the fiber direction was stretched significantly, indicating the near incompressibility of the myocardium. The results showed consistency across different hearts, with a variability of about 5% between experiments on different hearts. This may be attributed to physiological and anatomical differences.

Unlike Gupta et al. study where most of the attention is mainly based on the LV, this paper focuses on analysing the myocardium on both heart chambers, RV and LV, including the mid-wall (MDW) (Nemavhola, 2021a). The equi-biaxial test is performed on the two heart chambers (RV and LV) and MDW to determine the mechanical properties of the associated myocardia. Their (myocardia) mechanical properties are compared (in terms of stiffness) and discussed.

The mechanical property of the myocardium is normally acceptable to be used as a vital determinant of ventricular function (Laita et al., 2024; Liu and Wang, 2019). Moreover, myocardium mechanical properties can be useful for numerical cardiovascular predictions (Menon et al., 2024; Papamanolis et al., 2020; Kamaltdinov and Kuchumov, 2024; Khairulin et al., 2024; Kuchumov et al., 2023).

Despite the extensive use of sheep myocardium in cardiovascular research, a significant gap remains in the availability of detailed biaxial mechanical data, particularly regarding myocardial anisotropy and its implications for cardiac function. Previous studies have primarily relied on uniaxial testing, which, while informative, does not fully capture the complex, direction-dependent mechanical properties of myocardial tissue. Given that the heart undergoes multidirectional deformations during the cardiac cycle, understanding the biaxial behavior of the myocardium is crucial for accurately modeling its physiological and pathological states.

The present study addresses this gap by characterizing the passive mechanical behaviour of sheep myocardium in the LV, MDW and RV using biaxial extension testing. By examining cross-directional variations in myocardial mechanics and evaluating stored strain energy, we provide a more comprehensive understanding of myocardial anisotropy. Our approach offers improved precision over uniaxial tests and enhances the reliability of biomechanical models used for pathology evaluation and treatment prediction. This data is essential for refining computational models of the heart, improving surgical planning, and optimising therapeutic interventions for cardiovascular diseases.

## 2 Materials and methods

### 2.1 Tissue preparation

Healthy Sheep hearts were prepared (N = 10)with unknown heart conditions and age collected from a local abattoir a couple of hours after slaughtering. The visibly abnormal specimens were excluded to ensure that variability was minimised (a total of thirteen hearts were collected from the abattoir). Sheep myocardium is widely used as a model for human cardiac research due to its anatomical and mechanical similarities to human heart tissue. Both sheep and human myocardium exhibit comparable elastic modulus, stress-strain behavior, and contractile properties, making sheep a valuable proxy for studying cardiac biomechanics. The myocardial stiffness in sheep closely approximates that of humans, ensuring physiological relevance in studies related to heart failure, valve replacement, and myocardial infarction. The fiber orientation and ventricular structure in sheep hearts resemble those in humans, contributing to similar mechanical responses during cardiac cycles. These similarities make sheep an effective model for investigating therapeutic interventions, surgical techniques, and biomaterial testing in cardiovascular research (DiVincenti et al., 2014; Duchenne et al., 2019).

**FIGURE 1:**
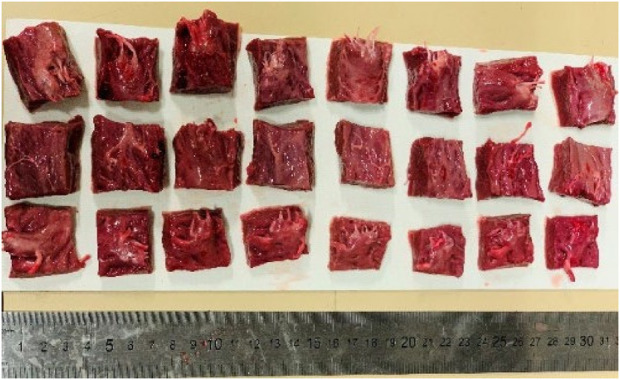
Preparation of the samples (sheep hearts) for mechanical testing

During the collection, hearts were placed in a cooler box and transported to the Biomechanics Lab. Immediately after arrival, the sheep hearts were prepared by being stored in a 0.9% NaCl saline solution for 30 min to ensure readiness for the biaxial testing. Once this was done, the sheep hearts were prepared by cutting a square of 18 mm × 18 mm (Figure 1).

### 2.2 Biaxial mechanical testing

**FIGURE 2:**
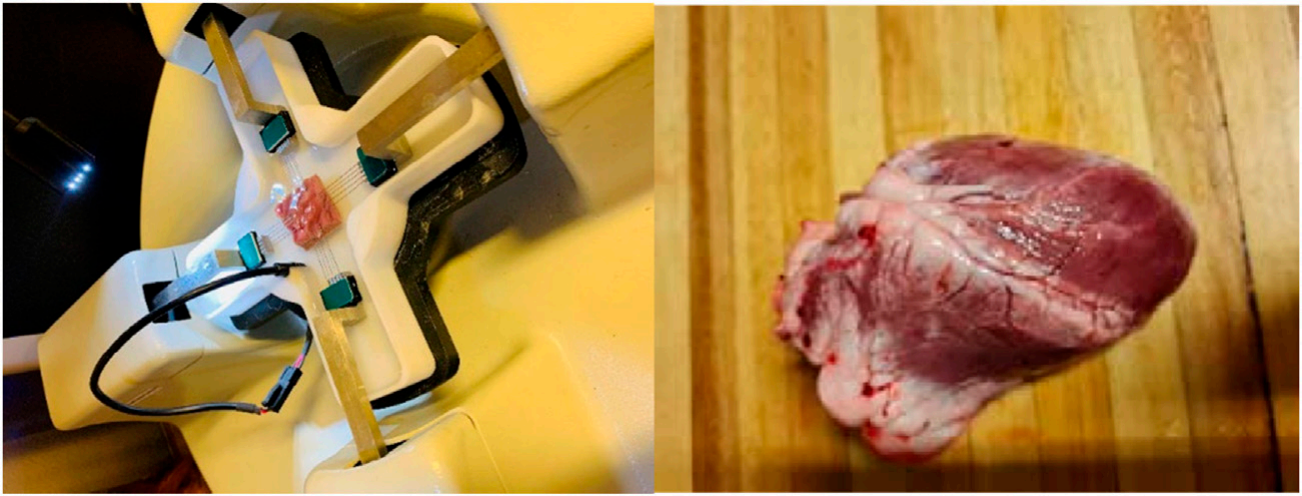
Experimental set up of equi-biaxial mechanical testing of sheep heart looking at three different regions including Left ventricle (LV), mid-wall (MDW) and Right ventricle (RV).

The biaxial testing methodology used, and protocol followed was mainly adopted from the previous work from the research group (Nemavhola, 2021a). The direction from the base to the apex was used and isolated papillary muscles as the main direction, the 18 mm × 18 mm square sample were cut from the left ventricle, right ventricle, and the septal wall. In this work, the longitudinal direction, which is along the papillary muscle, was used with the corresponding circumferential direction at 90° of the longitudinal direction. All the samples (LV, RV and MDW) were clapped using the Bio tester 5,000 CellScale, Waterloo Canada apparatus with approximately 16 mm × 16 mm ldimensions. The 0.9% NaCl saline solution (PSS) was heated to 37°C to mimic the body temperature before biaxial mechanical testing. A preload of 0.5 mN was then applied in each sample during testing and to reduce the built-in stress, all samples were subjected to a preconditioning as described in previous studies (Nemavhola, 2021b). Each sample was loaded to 40% strain equally in longitudinal and circumferential directions (Figure 2). The cross-sectional area of the samples was determined by measuring the thickness of the sample using a Vernier caliper. Ten (10) cyclic loading tests were considered for preconditioning to minimise the viscoelastic effects before the actual measurements. While we applied cyclic loading prior to the actual tests to mitigate viscoelastic effects and stabilise the tissue’s mechanical response, these may not fully capture the rate-dependent behavior of the myocardium (Anssari-Benam et al., 2019a; Anssari-Benam et al., 2020).

## 3 Experimental results

### 3.1 Stress-strain relationship

**FIGURE 3:**
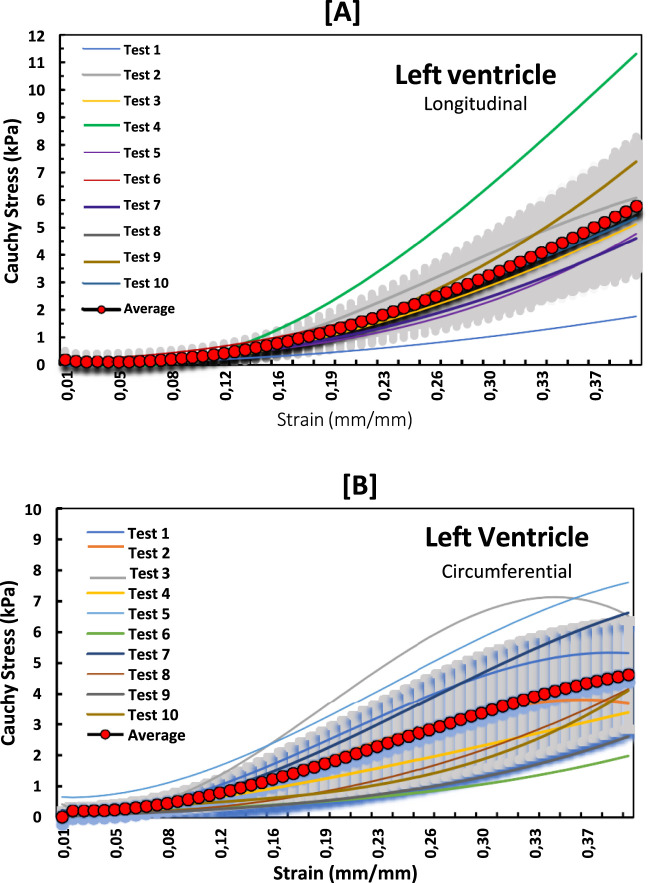
Stress-strain curves for LV myocardium in the **(A)** longitudinal and **(B)** circumferential directions overlayed with an average curve and standard error region.

**FIGURE 4:**
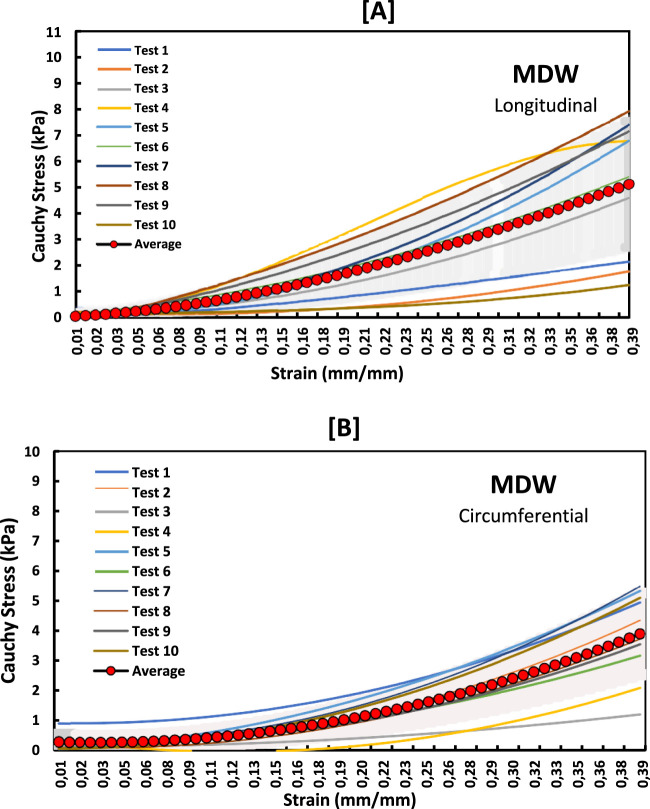
Stress-strain curves for MDW myocardium in the **(A)** longitudinal and **(B)** circumferential directions overlayed with an average curve and standard error region.

**FIGURE 5:**
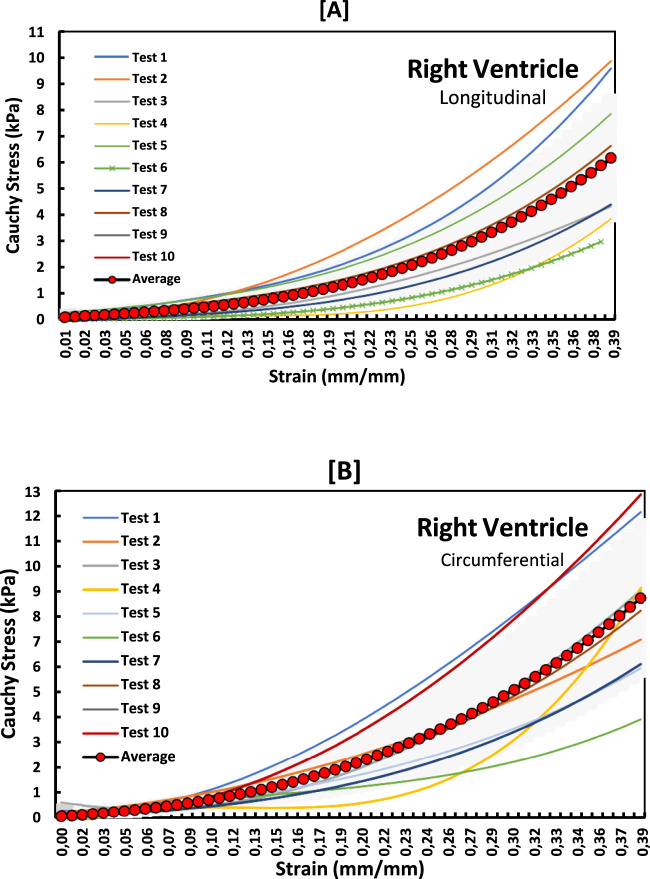
Stress-strain graphs for RV myocardium in the **(A)** longitudinal and **(B)** circumferential directions overlayed with an average curve and standard error region.

The non-linear stress-strain curves in Figures 3–5 are for the RV, MDW and LV, where a total of ten tests are for either longitudinal or circumferential direction. The curves are plotted up to a 40% strain rate in both longitudinal and circumferential directions. Though some of the stress-strain curves exhibit diminished toe regions probably due to differences in the preconditioning, the results show that the toe regions extend up to 16% strain rate for all the walls (Ngwangwa et al., 2022). The peak stress at the toe region (16% strain) marks a transition to a different mechanical phase; this may be attributed to fiber recruitment and collagen alignment. In addition, average curves have been added over the same axes for all the walls in both directions, and they fit in the middle of the other curves. The average curves are bound with the standard errors to show how well the ten test results fall within the standard deviation boundaries. Almost all curves (>6) fit within the one-standard deviation, except the MDW (longitudinal) with 50% lying outside the error bounds up until the end of the linear regions. According to statistics, 68% of the curves should be within the one-standard deviation boundaries which agrees with the current stress-strain curves for all the walls (Lane, 2013; Montgomery and Runger, 2007; Nemavhola et al., 2022). This proves the consistency and of the experimental procedure used and the quality of the acquired data in this study. The slight differences between the test results may have come from uncontrollable issues like poor clamping (loosening), differing conditions of animal sacrifice, different preloads histories (Ngwangwa et al., 2022).

**FIGURE 6:**
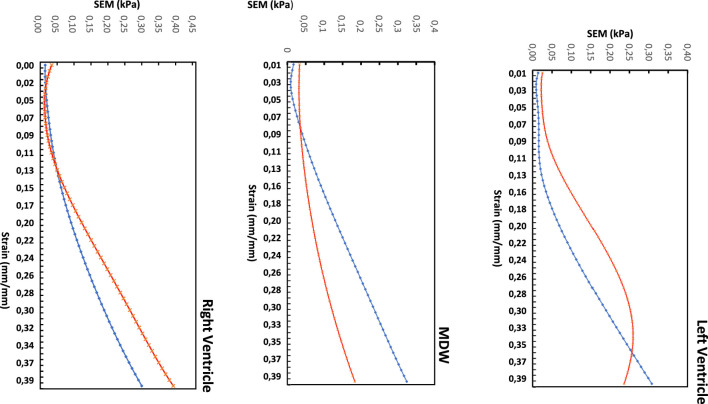
Standard error of the mean (SEM) for the three heart walls along each direction.

To examine the exact trend of the stress-strain curves relative to the average curves, a standard error of the mean (SEM) was calculated and plotted in Figure 6 below. The SEM results also show that the errors from the average curve increase with increased strain in the linear region for both longitudinal and circumferential directions in all the three walls. Among the three walls, the largest SEM occurs in the RV wall in the circumferential with SEM (0.39 kPa), whereas the longitudinal dominates in MDW and again circumferential dominates in the LV wall.

### 3.2 Cross-directional variation

**FIGURE 7:**
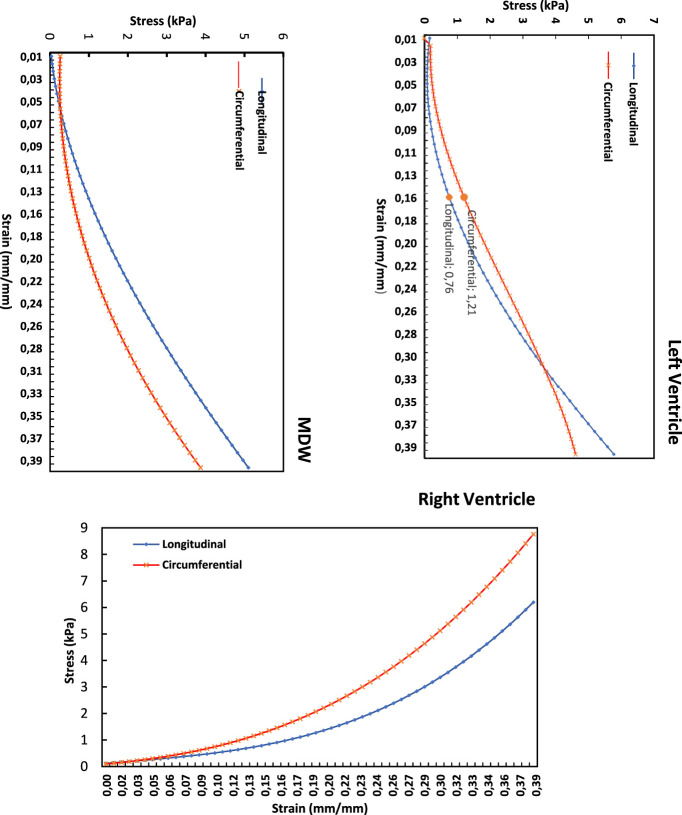
Longitudinal vs. circumferential stress-strain curves for each heart wall’s myocardium.

Figure 7 shows the average stress-strain curves along the longitudinal and circumferential directions plotted on the same LV, MDW and RV axes. For the RV and MDW, the myocardium exhibits qualitatively similar trends, although the circumferential has the highest stress (8.8 kpa) and dominates on the RV, unlike on the MDW, where the longitudinal dominates throughout. The LV exhibits a unique qualitative trend as the circumferential moves very close-by the longitudinal but dominates first along the first three-quarters of the curve and then get overlapped at the peak. The tissues are, on average, more compliant (flat) along the longitudinal than the circumferential direction in the toe region. In the linear region, however, the RV and MDW have the same longitudinal and circumferential stiffness. The LV has a higher stiffness along the longitudinal compared to the circumferential.

These differences are further qualitatively evaluated in this study by using the single-factor analysis of variance. The Shapiro–Wilk test was conducted which is more appropriate method for small sample sizes. Most tests for Strain and Strain had p-values >0.05, meaning the data is normally distributed in several test groups (Cohen, 2013). Table 1 summarises the results regarding the cross-directional variation of elastic modulus, peak stress at toe region limits and stress at 40% strain for each wall. The elastic modulus was determined from the linear elastic region for each heart wall specifically, from 16% to 40% strain for LV, MDW, and RV. The toe region peak stresses were those corresponding to 16% strain for LV, MDW, and RV. The stresses at 40% strain were obtained from the closest strain level. It is assumed that for a displacement-controlled test such as the one used in this study, it makes more sense to monitor the differences in the peak stresses than in the strains. The peak stresses are therefore used to reveal some nuances in the differences in the material behaviour during the straining process (Krüger, 2024).

**TABLE 1:** Statistical significance test results for axis-to-axis variations in biaxial mechanical parameters.

	P-values: Cross-directional variation in a wall		
	LV	MDW	RV
Elastic modulus	0.065	0.314	0.067
Peak stress at toe region limit	0.12	0.089	0.046
Peak stress at 40% strain	0.293	0.188	0.051

The results show that the MDW of sheep myocardium exhibits statistically highest (non-significant) variation in elastic modulus between the longitudinal and circumferential directions. For LV and RV the elastic modulus variation is light but analyses of variance in the stresses yield significant variances in tissue responses at the toe region limit for the RV, and both at the toe region limit and at 40% strain for the RV.

**FIGURE 8:**
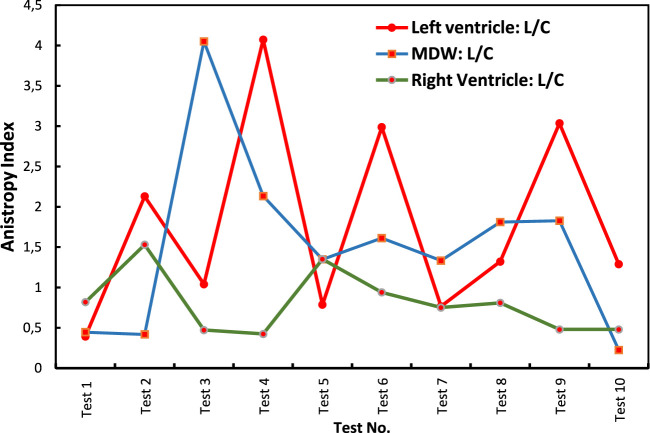
Anisotropy indices calculated over all tests for each heart wall.

Figure 8 shows indices of anisotropy which are calculated by relating tissue elastic moduli in the longitudinal and circumferential directions for each test. The results show that the LV has the highest anisotropy averaged at around an index of 1.8. The MDW and RV have average anisotropy indices of 1.5 and 0.8, respectively. Research on decellularized rat RV tissue found an average anisotropy index of 0.71 in the central region, indicating the tissue is approximately five times stiffer in the preferred direction (Witzenburg et al., 2012). Regarding the mid-distal wall (MDW), specific anisotropy index values are not readily available in the current literature. However, it's known that different regions of the heart exhibit unique anisotropic properties due to variations in myocardial fiber orientation and structural composition. For example, studies have shown that the distal region of certain tissues is stiffer than the middle region and exhibits stiffness anisotropy (Zhu et al., 2020).

### 3.3 Cross-wall variation

The tissue behaviour in the linear region differs for all three wall tissues. The elastic moduli and averaged stress values for the three walls in the longitudinal and circumferential directions are shown in Table 2 below. These results show that this study’s RV and LV myocardial tissues are stiffer than the MDW myocardial wall in both directions. The RV myocardial tissue is stiffest in the linear region in both directions. However, it is important to note that this is not the case in the toe region where the LV myocardial tissues are on average the second stiffest in both directions.

**TABLE 2:** Average elastic moduli, stress at toe and stress at 40% across all the walls and both directions.

	LV		MDW		RV	
	L	C	L	C	L	C
Elastic moduli (E)	20.7	14.2	16.34	13.2	22	30.5
Stress at toe region	0.85	1.3	1.2	0.7	0.9	1.45
Stress at 40%	6 ± 2.3	5 ± 1.75	5 ± 2.4	4 ± 1.4	6 ± 2.3	9 ± 3

L and C, longitudinal and circumferential.

Table 3 summarises the statistical significance tests for cross-wall variations of elastic moduli, toe region peak stresses, and stress at 40% strain for each direction. For elastic moduli, the results show that the only non-significant differences occur between the LV and RV along the longitudinal direction and again between LV and MDW along the circumferential direction. The rest of the cross-wall relationships yield significant differences for the elastic moduli. The peak stress at the toe region does not yield any significant difference between the LV and RV along the longitudinal direction. The results in the table show that from one wall to another, the most significant differences occur along the circumferential direction. This shows that each wall of the sheep heart has distinct mechanical properties since there is at least one parameter (longitudinal) that is different from wall to wall.

**TABLE 3:** Statistical significance test results for cross-wall variations in biaxial mechanical parameters.

	P-values: Cross-wall variation in a particular direction					
	LV-MDW		RV-LV		RV-MDW	
	L	C	L	C	L	C
Elastic Modulus	0.276	0.7	0.76	0.0000	0.14	0.0000
Peak stress at toe region limit	0.15	0.072	0.77	0.66	0.268	0.0082
Peak stress at 40% strain	0.58	0.29	0.75	0.0029	0.387	0.0000

L and C, longitudinal and circumferential.

**FIGURE 9:**
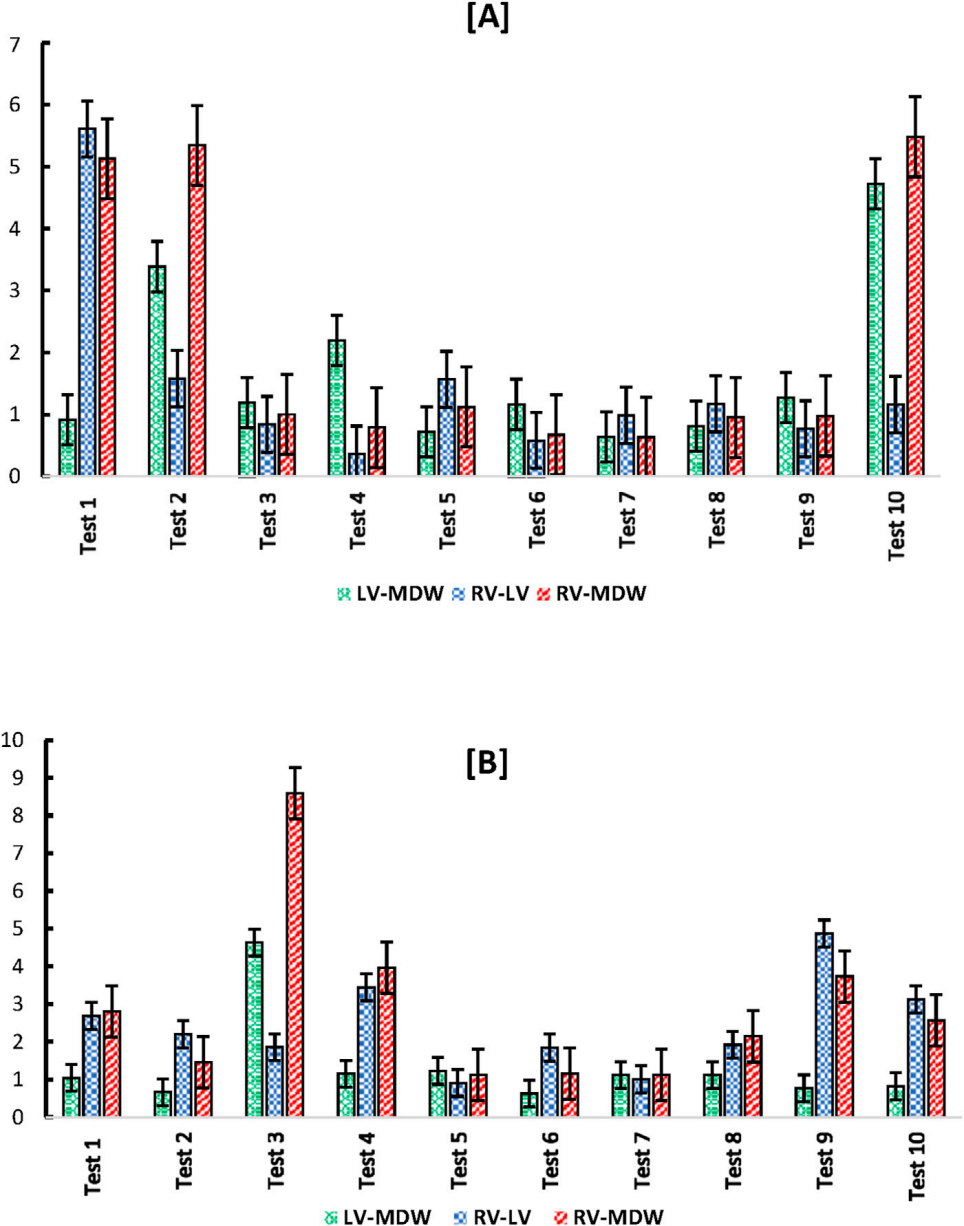
Cross-wall **(A)** longitudinal-to-longitudinal, and **(B)** circumferential-to-circumferential ratios between different heart walls for each test.

**FIGURE 10:**
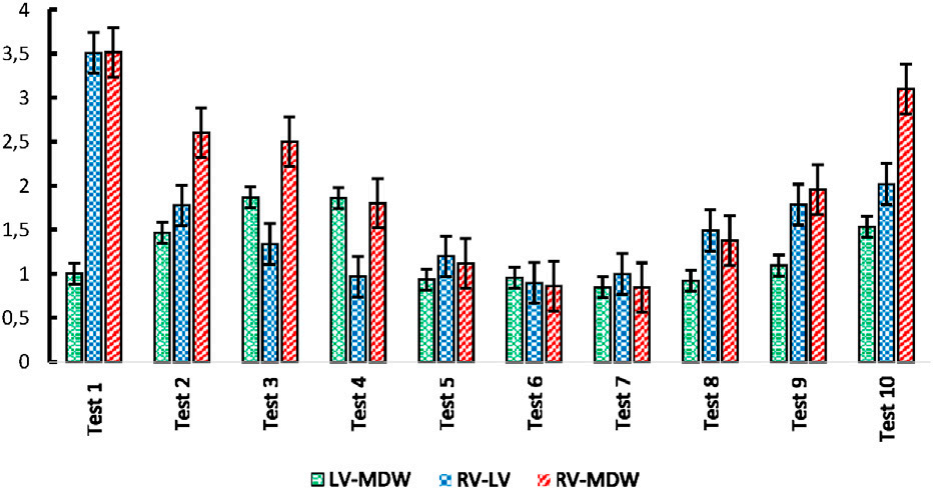
Index of anisotropy for all tests.

Ratios of cross-wall elastic moduli for all tests (one by one) are shown in Figure 9. The graphs clearly show that the heart walls are distinct from each other in either direction. There is no ratio that lies close to unity for all cross-wall relationships shown in Figures 9, 10

### 3.4 Stored strain energy

The passive response of the tissues can be linked to the amount of strain energy that can potentially be stored under load. In this study the stored strain energy in the toe and linear regions along both directions were evaluated and tabulated in Table 4. The stored strain energy is numerically calculated using the trapezoidal rule given by Equation 1.

$$U=\sum_{n=1}^{n-1}\frac{1}{2}\sigma^{n+1}+\sigma^{n}\times \varepsilon^{n+1}-\varepsilon^{n}$$ (1)

**TABLE 4:** Stored strain energy values calculated within the toe region and linear elastic region for each heart wall myocardium.

Wall of myocardium	Toe region (kJ)		Linear elastic region (kJ)	
	Longitudinal	Circumferential	Longitudinal	Circumferential
Left ventricle	0.53	1.02	7.317	6.67
Mid-wall	0.99	0.76	7.065	5.072
Right ventricle	0.8	1.21	7.76	11.25

Where n is a sample point and N is the total length of the measured data.

The results in Table 4 show that the RV stores the largest amount of strain energy along both directions. Due to its low stiffness values at linear regions and low peak stresses at 40%, the MDW myocardium does have the least amount of stored strain energy except along the longitudinal direction in the toe region. According to this study the RV stores much higher strain energy than the LV in the linear region, and LV stores more strain energy than the MDW. The high strain energy on the RV shows that the wall can be of assistance (plan B) to the LV in pumping the blood to the rest of the body.

## 4 Discussion

The mechanical properties of myocardium tissues are crucial in understanding cardiac function and potential failure mechanisms. This study focuses on the passive biaxial mechanical properties of sheep myocardium tissues from different regions of the heart: the left ventricle (LV), mid-wall (MDW), and right ventricle (RV).

The mechanical behavior of cardiac tissues is inherently rate-dependent due to their viscoelastic nature, which arises from the complex interplay between extracellular matrix (ECM) components such as collagen, elastin, glycosaminoglycans, and proteoglycans (Anssari-Benam et al., 2019b). These constituents play a crucial role in governing the time-dependent response of myocardial tissue to mechanical loading. Collagen provides structural integrity and tensile strength, elastin allows for elasticity and recoil, and glycosaminoglycans contribute to the tissue’s ability to retain water, further modulating its viscoelastic behavior. The dynamic response of these components to strain rates results in variations in stiffness, energy dissipation, and stress relaxation, all of which are critical in understanding the biomechanics of cardiac tissue.

In this study, we conducted mechanical tests at a single strain rate to maintain consistency across samples, ensuring uniform conditions for comparison. However, previous studies have demonstrated that non-preconditioned cardiac tissues exhibit rate-dependent mechanical responses, with differences in stress-strain behavior, hysteresis, and relaxation properties when tested at varying strain rates (Anssari-Benam et al., 2018). This rate dependence is particularly relevant for understanding physiological and pathological conditions, as alterations in ECM composition such as fibrosis or degradation can significantly impact the tissue’s mechanical response to different loading rates.

### 4.1 Stress-strain behavior

The non-linear stress-strain curves for the LV, MDW, and RV revealed significant differences in mechanical behavior across these regions. The LV myocardium demonstrated higher stiffness in both the longitudinal and circumferential directions compared to the RV and MDW. The toe region, which represents the initial, more compliant phase of tissue deformation, extended up to 16% strain for all regions. However, beyond this point, the LV exhibited the highest stiffness, particularly in the longitudinal direction, suggesting a greater ability to withstand higher stress levels (Borlaug et al., 2013).

The RV myocardium, on the other hand, showed the highest stored strain energy, indicating a higher capacity for deformation before reaching its elastic limit. This is particularly relevant in the context of the RV’s role in accommodating varying volumes of blood during cardiac cycles. The MDW myocardium displayed intermediate properties, with less stiffness and lower stored strain energy compared to the LV and RV (Erley et al., 2020; Liu et al., 2022).

### 4.2 Anisotropy and cross-directional variation

The study found that the LV exhibited the highest anisotropy index, indicating a significant difference in stiffness between the longitudinal and circumferential directions (Liu et al., 2021; Goetz et al., 2024; Corporan et al., 2022). This anisotropic behavior is crucial for the LV’s function, as it needs to generate sufficient pressure to pump blood throughout the body (Sacks et al., 2009). The RV and MDW showed lower anisotropy indices, suggesting more uniform mechanical properties in different directions (Liu et al., 2023).

Cross-directional variations in stress-strain behavior were also statistically significant, particularly in the RV and LV. The RV showed the most significant variance in elastic modulus between the longitudinal and circumferential directions, highlighting the mechanical adaptations required to handle varying blood volumes and pressures.

### 4.3 Cross-wall variation

Cross-wall analysis revealed distinct mechanical properties for each myocardium region. The RV was the stiffest in the linear region, suggesting a robust structure capable of enduring high stress levels during contraction. However, in the toe region, the LV was second stiffest, indicating a balance between compliance and stiffness necessary for efficient blood ejection. The MDW was the least stiff, potentially due to its location and function within the heart structure (Novak et al., 1994). Statistical analyses confirmed significant differences in mechanical properties between the myocardium regions, particularly in elastic moduli and peak stresses at different strain levels. These differences underline the specialized roles of each heart chamber and their adaptations to specific functional demands.

### 4.4 Stored strain energy

The stored strain energy, a measure of the tissue’s ability to store elastic energy, was highest in the RV, particularly in the linear region. This property is crucial for the RV’s role in handling large volumes of blood. The LV stored more strain energy than the MDW, reflecting its critical function in generating high pressures for systemic circulation. These findings suggest that the mechanical properties of the myocardium are finely tuned to the functional requirements of each heart region.

## 5 Conclusion

This study provides a comprehensive analysis of the passive biaxial mechanical properties of sheep myocardium, highlighting significant differences across the LV, MDW, and RV. The LV exhibits the highest stiffness and anisotropy, reflecting its role in high-pressure blood ejection. The RV shows the highest stored strain energy, essential for accommodating varying blood volumes. The MDW displays intermediate properties, suggesting a balancing role within the heart structure.

Understanding these mechanical properties is crucial for developing better models of heart function and failure. It also provides valuable insights for designing targeted therapies and interventions for cardiac diseases. Future research should explore the implications of these findings in pathological conditions and extend the analysis to other species to generalize the mechanical behavior of myocardium tissues. For enhanced clinical relevance, the investigation of myocardial disease states, including porcine or human myocardium, can be considered in future research.

## 6 Limitations

• The effect of age on the mechanical properties of the myocardium was not considered. Future studies should consider investigating a range of loading rates to better understand the rate-dependent mechanical properties of cardiac tissues.
• Another limitations of this study is the small sample size, which may impact the generalisability of our findings. Given the exploratory nature of this research, power calculations were not performed, potentially limiting the statistical strength of our conclusions. While our results provide valuable insights into the biaxial mechanical behavior of sheep myocardium, a larger sample size would improve the robustness of the data and allow for a more comprehensive assessment of inter-subject variability.

## Data Availability

The original contributions presented in the study are included in the article/supplementary material, further inquiries can be directed to the corresponding author.
